# EXAMINING CAREGIVER NETWORK STRUCTURE, COMPOSITION, AND COORDINATION FOR OLDER ADULTS WITH ALZHEIMER’S DISEASE

**DOI:** 10.1093/geroni/igad104.0060

**Published:** 2023-12-21

**Authors:** William McConnell, Katherine Haggar, María Ortega Hernández

**Affiliations:** Florida Atlantic University, Boca Raton, Florida, United States; Florida Atlantic University, Boca Raton, Florida, United States; Florida Atlantic University, Boca Raton, Florida, United States

## Abstract

Over half of the older adults with Alzheimer’s disease and related dementias (ADRD) live in the community with informal caregivers whose unpaid assistance constitutes the majority of their care. Research and interventions targeting caregivers typically limit their focus to one primary caregiver, but many older adults with ADRD rely on a variety of interconnected kin, non-kin, and professional supporters. Little is known about the structure, composition, or consequences of these caregiver networks. To address this gap, we conducted a social network analysis (SNA) of the caregiver networks of 23 older adults with ADRD receiving outpatient services at a Memory Disorder Clinic. SNA data was obtained through detailed social network interviews conducted one-on-one with self-identified primary caregivers. We find that adults with ADRD have an average of 7.7 caregivers in their networks. Quantitative analyses demonstrate that caregiver network properties (including size, density, composition, and care coordination) vary with the context of caregiving (including living situation, level of functional impairment, and use of formal services). An average network includes a roughly even mix of informal caregivers (3.2 members) and professional caregivers (3.5), but they occupy different network positions and fill distinct supportive roles. For example, informal caregivers engage in frequent care coordination, but coordination is comparatively uncommon between formal caregivers. This interdisciplinary research advances beyond the dyadic level of analysis typical of most caregiving research to comprehensively measure caregiver networks. Further, we identify variations in caregiver networks and quantify network-based task-sharing and care coordination among informal and professional caregivers.

